# The function and regulation of the bHLH gene, *cato*, in *Drosophila *neurogenesis

**DOI:** 10.1186/1471-213X-10-34

**Published:** 2010-03-26

**Authors:** Petra I zur Lage, Andrew P Jarman

**Affiliations:** 1 Centre for Integrative Physiology, School of Biomedical Sciences, University of Edinburgh, Edinburgh EH8 9XD, UK

## Abstract

**Background:**

bHLH transcription factors play many roles in neural development. *cousin of atonal *(*cato*) encodes one such factor that is expressed widely in the developing sensory nervous system of *Drosophila*. However, nothing definitive was known of its function owing to the lack of specific mutations.

**Results:**

We characterised the expression pattern of *cato *in detail using newly raised antibodies and GFP reporter gene constructs. Expression is predominantly in sensory lineages that depend on the *atonal *and *amos *proneural genes. In lineages that depend on the *scute *proneural gene, *cato *is expressed later and seems to be particularly associated with the type II neurons. Consistent with this, we find evidence that *cato *is a direct target gene of Atonal and Amos, but not of Scute. We generated two specific mutations of *cato*. Mutant embryos show several defects in chordotonal sensory lineages, most notably the duplication of the sensory neuron, which appears to be caused by an extra cell division. In addition, we show that *cato *is required to form the single chordotonal organ that persists in *atonal *mutant embryos.

**Conclusions:**

We conclude that although widely expressed in the developing PNS, *cato *is expressed and regulated very differently in different sensory lineages. Mutant phenotypes correlate with *cato's *major expression in the chordotonal sensory lineage. In these cells, we propose that it plays roles in sense organ precursor maintenance and/or identity, and in controlling the number of cell divisions in the neuronal branch of the lineage arising from these precursors.

## Background

Basic-helix-loop-helix (bHLH) transcription factors are central to neurogenesis in metazoans [1]. The most well known role for such factors in neurogenesis is the so-called 'proneural' function. This function underlies the commitment of neuroectodermal cells to a neural fate, and the term comes originally from the study of proneural genes in *Drosophila*. In this organism, proneural genes include *atonal *(*ato*), *amos*, *scute *(*sc*), and *achaete *(*ac*) which are required for the specification of sense organ precursors (SOPs) of the peripheral nervous system [2]. In mutations of these genes, specific subsets of SOPs fail to be formed. For instance, *ato *is required for the formation of SOPs of chordotonal (Ch) proprioceptive sensory organs [3].

Other members of the bHLH protein family are expressed after neural commitment and play a variety of roles in neural cells leading up to neural differentiation. This is particularly apparent in vertebrates, where for instance the factors NeuroM and NeuroD are required for neuronal migration and differentiation respectively [4,5]. In *Drosophila*, such 'downstream' neural bHLH factors are represented by *asense *(*ase*), *cousin of atonal *(*cato*), *deadpan *(*dpn*) and *target of poxn *(*tap*). These genes are related to *sc*, *ato*, *hairy/E(spl) *and *neurogenin *respectively. *ase*, *cato *and *dpn *are widely expressed in developing neurons [6-8], whereas *tap *expression is confined to a small subset of sensory neurons [9]. The functions of these genes are less well known compared with proneural genes. *ase *is expressed in all neural precursors of both the CNS and PNS [6]. Mutations of *ase *result in reduced viability but mutant embryos exhibit only subtle PNS defects [10]. In the larval optic lobes, *ase *participates in the control of mitotic activity in neural precursors [11]. In this process, *ase *limits proliferation by antagonising *dpn*. In turn, *dpn *antagonises *dacapo *(*dap*) [12-14]. *dap *encodes a p21 cyclin-dependent kinase (CDK) inhibitor that is expressed transiently in cells prior to their terminal cell division in order to prevent further divisions [12-14].

Unlike *ase *and *dpn*, the expression of *cato *is confined to the developing PNS, where it was reported to be expressed in all SOPs and their progeny [7]. The function of *cato *is poorly known. Examination of embryos bearing large deficiencies of the *cato *region suggested a role in sensory neuron differentiation [7]. We report here the generation and analysis of specific *cato *mutations. Flies homozygous for *cato *loss-of-function mutations are viable. Mutant embryos show no gross neuronal differentiation defects, but have a defect in cell proliferation within the Ch sensory lineages. Combination of *cato *mutation with those of *ato *and *ase *reveals a second role for *cato *in the maintenance of Ch SOP fate or survival.

## Results

### Expression of Cato differs in Ato/Amos and Sc lineages

**Figure 1:**
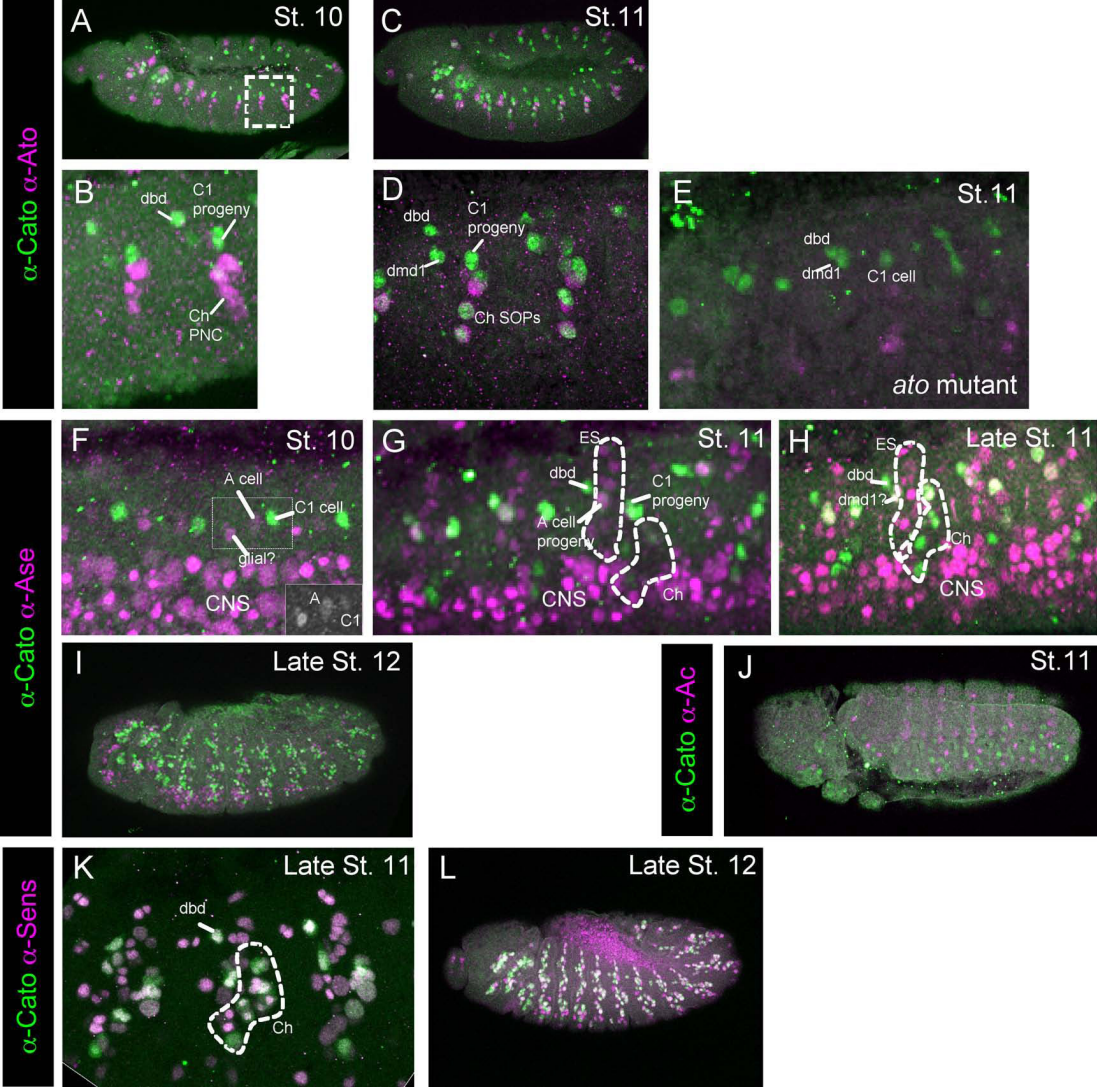
**Cato protein expression in the embryo**. Immunohistochemistry with anti-Cato (green) and a second marker antibody (magenta). (A-E) Expression of Cato relative to Ato. (A, B) Stage 10 embryo. Boxed area is magnified in (B). Cato is expressed in the C1 progeny, the dbd precursor and the probable dmd1 precursor; at this stage Ato is mostly in the proneural cluster (PNC) cells for the remaining Ch precursors. (C) Stage 11 embryo. (D) Magnification of two abdominal segments (different embryo to (C)). Cato is now expressed additionally in further Ch SOPs. (E) Stage 11 *ato*^1 ^mutant embryo, showing that Cato expression remains in the C1, dbd and dmd1 precursors. Note that non-functional Ato protein is still expressed in these embryos. (F-I) Cato expression relative to the SOP marker, Ase. (F) Stage 10 embryo, with Cato in C1. At this early stage Ase is weakly expressed in C1 and A cells, and a glial cell, and strongly expressed in neuroblasts of the CNS. The green channel for the region boxed is shown in the inset image. (G) Stage 11 embryo, showing more ES precursors (expressing Ase) but these do not express Cato at this stage. (H) Late stage 11 embryos, showing that Cato is still not expressed strongly in most ES cells (Ase-positive cells). (I) Late stage 12 embryo, Cato is now expressed widely in PNS cells. (J, K) Cato expression relative to Sens. At early (J) and late (K) stage 12 there is substantial co-expression of Cato and Sens in PNS cells.

It was previously reported that *cato *mRNA was initially activated in Ch SOPs, and subsequently it appeared to be expressed generally in all cells of the sensory PNS (both *ato*-dependent Ch cells and *sc*-dependent External Sensory (ES) cells) [7]. An anti-Cato antibody confirms and extends these findings. Expression of Cato protein was assessed relative to other markers of early sensory neurogenesis. These include: Amos, which marks the *amos*-dependent Dorsal Bipolar Dendrite (dbd) and Dorsal Multidendritic 1 (dmd1) neural precursors [15]; Ato, which marks all Ch precursors as well as the dbd and dmd1 cells [3,16]; and Ase and Senseless (Sens), which mark all SOPs and their progeny [6,17]. Cato protein is initially detectable at stage 10 in the first *ato*-dependent Ch SOP (known as C1 or the 'P' cell [18]), followed shortly by the first *amos*-dependent precursor, dbd (Fig. 1A, B, F). At this stage it is not expressed in the so-called 'A' cell, which is the first ES SOP to appear (Fig. 1F, G). Shortly after, it is expressed in further Ch SOPs and also in a cell presumed to be the second *amos*-dependent precursor, dmd1 [16] (Fig. 1C, D, H). At this stage, Cato is still not expressed in ES cells, although numerous ES cells are detectable by Ase and Sens expression (Fig. 1F-H). Eventually, at late stage 11/stage 12, Cato is detectable in the progeny of most SOPs, including ES cells, and overlaps extensively but not completely with Ase and Sens (Fig. 1I-K). In *ato *mutant embryos, Cato expression is reduced, consistent with loss of most Ch precursors (Fig 1F). However, expression remains in the Amos-dependent cells. It also remains in the C1 cell, consistent with the observation that this Ch precursor usually appears even in the absence of *ato *function [7,19] (Fig. 1E). In summary, Cato is first expressed in *ato*- and *amos*-dependent SOPs. In contrast, it is not expressed in *sc*-dependent ES cells until after the first division of the SOPs. This difference in expression between the two sensory lineages was also observed in imaginal discs (data not shown).

**Figure 2:**
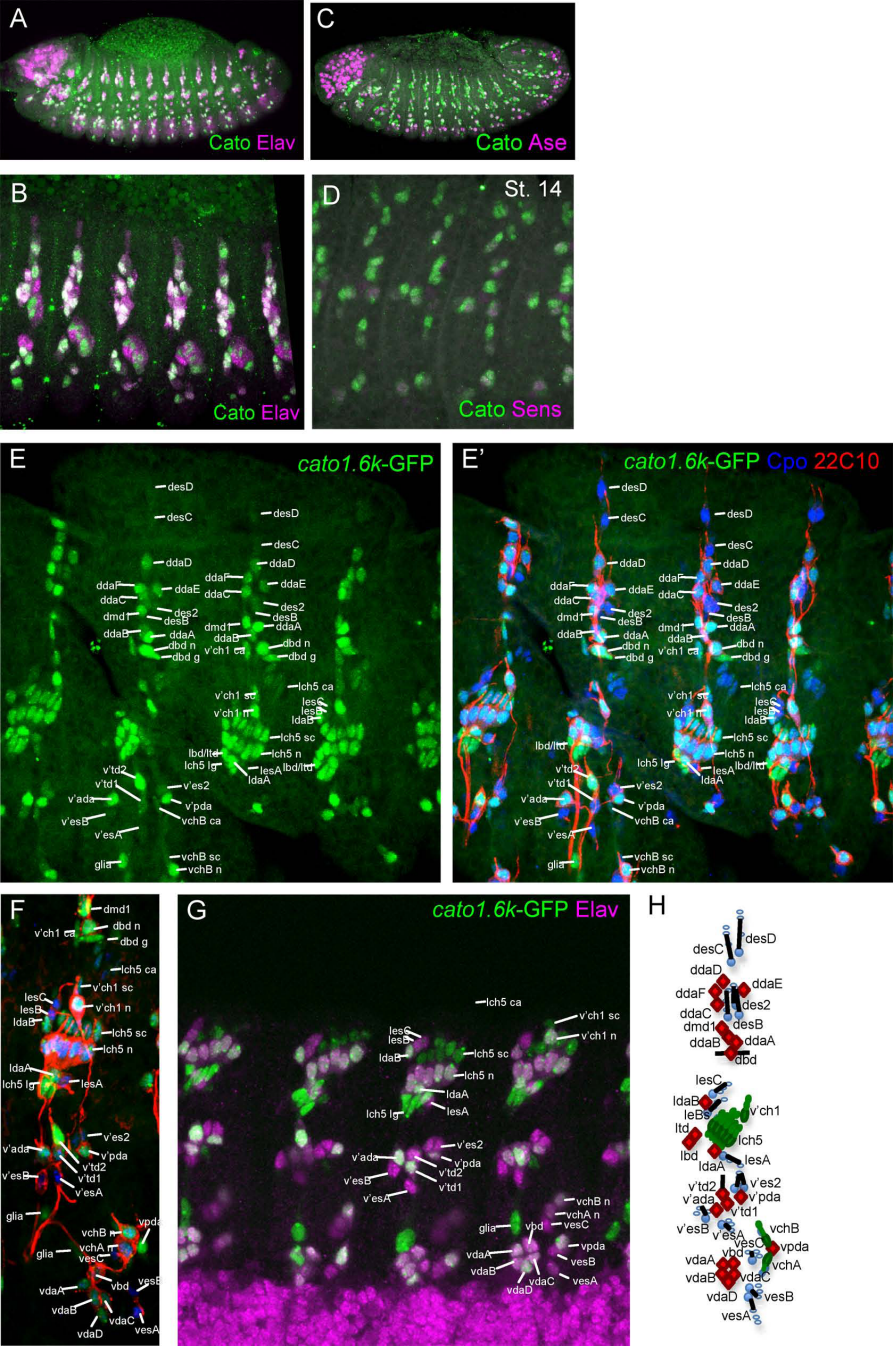
**Cato protein expression relative to late neural markers**. Immunohistochemistry with anti-Cato (green) and a second marker antibody (magenta). (A) Stage 14 embryo showing substantial co-expression of Cato and Elav, a neuronal differentiation marker. (B) Larger magnification view of similar embryo showing dorsolateral regions of several abdominal segments. (C) Stage 13 embryo, showing that Cato expression generally persists longer than Ase in most cells. (D) Ventrolateral view of stage 14 embryo, showing Cato expression generally more persistent that Sens. (E-G) Expression of the *cato1.6k*-GFP reporter gene. (E, E') Stage 16 embryo, dorsolateral abdominal segments, showing GFP expression (green) relative to Cpo, which marks nuclei of all PNS cells (blue) and 22C10, which marks sensory neurons (red). (F) Ventrolateral view of a similar embryo. (G) Stage 16 embryo showing expression of GFP relative to Elav, ventrolateral view of abdominal segments. (H) Schematic representation of sensory neurons (filled) and support cells (unfilled) of an abdominal segment (based on [44]): Neurons are: Ch (green), ES (blue); md (red). Abbreviations: v = ventral; v' = ventral'; l = lateral; d = dorsal; ch = chordotonal; es = external sensory; da = dendritic arborisation neuron; bd = bipolar dendritic neuron; td = tracheal dendritic neuron; md = multidendritic neuron; n = neuron; sc = scolopale cell; lg = ligament cell; ca = -cap cell; g = glial cell.

Cato protein expression persists in sensory lineages until terminal differentiation. Expression ceases only after the beginning of terminal differentiation, since there is substantial co-expression of Cato and Elav, a terminal differentiation marker (Fig. 2A, B). The latest expression appears to mark a specific subset of cells that only partially overlaps with the expression of Ase and Sens (Fig. 2B-D). This later expression is recapitulated by a GFP reporter gene construct driven by the genomic region upstream of *cato*, which contains enhancers for the entire *cato *expression pattern (see below; S. Cachero, PzL, APJ, submitted). Between stages 10 and 14, GFP expression reflects that of endogenous Cato (except for the delay associated with GFP maturation). In Ch lineages, GFP perdures strongly into neuron, scolopale and ligament cells, but only weakly in cap and attachment cells (Fig. 2E-H). This suggests that whilst *cato *expression begins in the Ch SOPs, it is subsequently maintained preferentially in daughter pIIb and then pIIIb - the intermediate precursors leading to the neuronal branch of the sense organ lineage [20]. Expression also perdures in the dbd neuron and its glial sib, in one of the v'td neurons, and in a possible peripheral glia cell (Fig. 2E-H). In ES organs, GFP is only weakly expressed in general. However, it perdures strongest in most of the multidendritic (md) neurons that derive from ES lineages (in addition to those derived from Ch lineages) (Fig. 2E-H). This suggests that the late expression of Cato protein noted above is mostly associated with Ch and md neurons.

### Evidence that Cato is directly regulated by Ato and Amos but only indirectly regulated by Sc

Cato is expressed in *ato*- and *amos*-dependent SOPs, where its expression overlaps that of Ato and Amos (Fig. 1A-D and data not shown). In contrast, Cato is not expressed until later in the progeny of Sc-dependent ES SOPs. Sc expression in ES SOPs is transient: it is downregulated before SOP division [21]. Cato expression does not begin until after this time. It therefore appears that Cato expression does not overlap with that of Sc in the ES lineage. Consistent with this, at stage 11 ES cells express the direct Sc targets, *ase *and *sens*, but not *cato *(Fig. 1F-H). Moreover, Cato expression does not overlap with the expression of the proneural gene, Ac, which is thought to be coexpressed with Sc [22] (Fig. 1J). Therefore, as a proneural target gene, *cato *may be directly regulated by Ato and Amos but only indirectly regulated by Sc.

**Figure 3:**
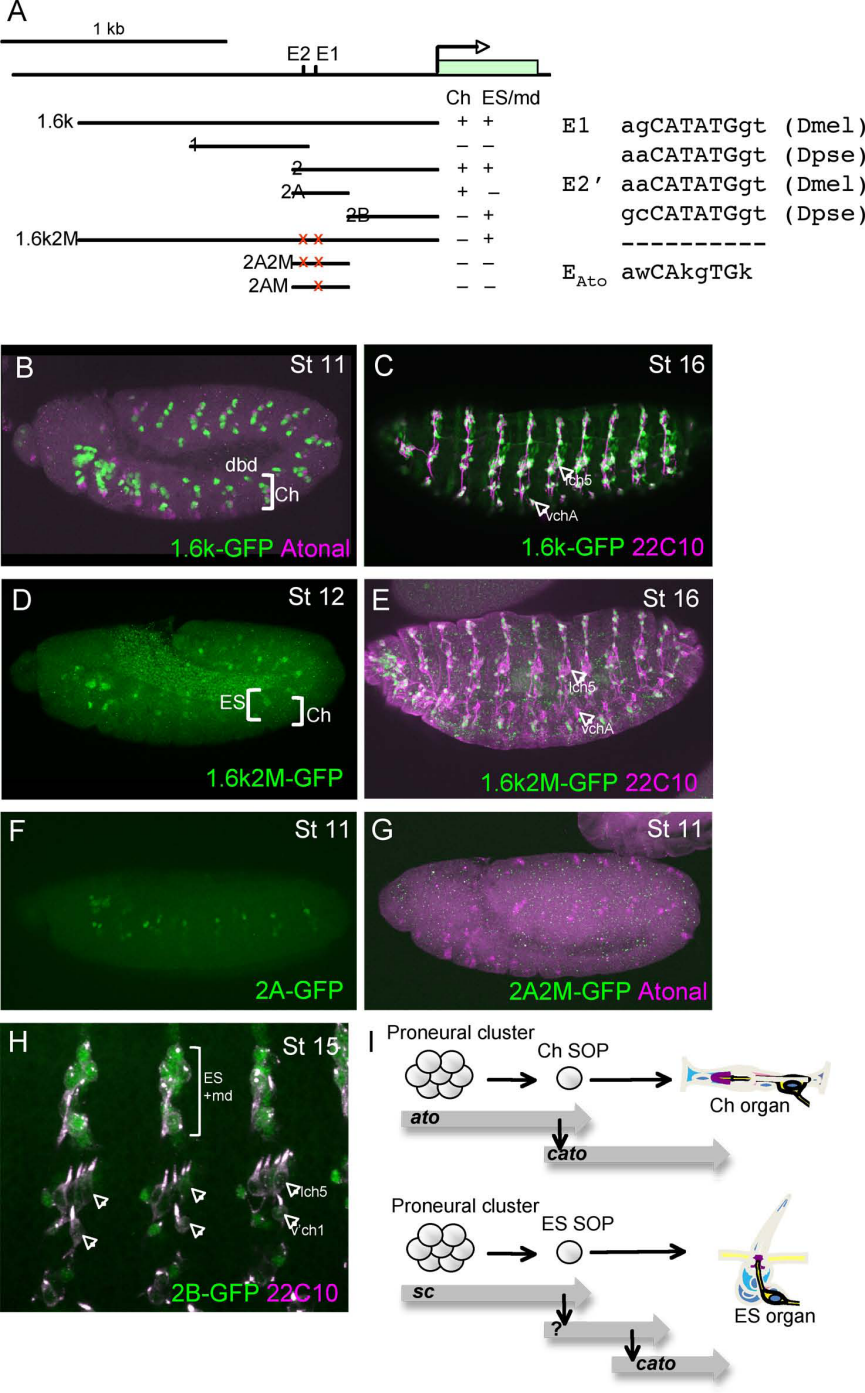
**Cato has separate Ch and ES/md enhancers**. (A) Schematic showing the upstream region of *cato*, the fragments tested by GFP reporter gene analysis, and a summary of the expression patterns supported (Ch or ES/md). The positions of the two E boxes mentioned in the text are shown, as are their sequences in *D. melanogaster *and *pseudoobscura *compared to the known Ato consensus binding site. (B-H) GFP expression in embryos containing different *cato *reporter genes (green). (B)*cato1.6k*-GFP in early stage embryo showing early expression in Ch and dbd cells. (C) *cato1.6k*-GFP in late stage embryo showing expression in all Ch lineages (arrows) and da neurons (costained with 22C10 in magenta). (D) *cato1.6k2 M*-GFP in stage 12 embryo showing expression just beginning in some ES cells but loss of expression in Ch lineages. (E) *cato1.6k2 M*-GFP in late stage embryo showing expression in da neurons but loss of expression in Ch lineages (arrows). (F) *cato2A*-GFP in early embryo showing early expression in Ch precursors (G) *cato2A2 M*-GFP expression in early embryo, showing lack of Ch expression. (H) *cato2B*-GFP in late embryo showing expression non-Ch lineages but not in Ch lineages (arrows). (I) Summary of expression pattern and reporter gene evidence that suggests that *cato *is a direct target of Ato (and Amos), but an indirect target of Sc.

To investigate this, we identified the *cis*-regulatory elements of *cato *(Fig. 3A). A 1.6-kb genomic DNA fragment upstream of the *cato *transcription unit supports GFP reporter expression in a pattern closely resembling that of the endogenous gene. That is, expression is observed initially in Ch cells and later in all PNS cells (Fig. 3B, C). Examination of the sequence of the 1.6-kb region revealed the presence of several E box motifs that resemble the previously identified Ato-specific DNA binding site (EAto)[23](Fig. 3A). When two of these EAto boxes were mutated, the early expression of GFP in Ch cells was abolished but later ES/md cell expression was unaffected (Fig. 3D). Subsequently, we found that the 1.6-kb fragment could be subdivided into at least two enhancers, one that supports early expression in *ato*- and *amos*-dependent cells (*cato2A*; Fig. 3E, F) and one that shows late expression in ES/md cells (*cato2B*; Fig. 3H). The former contains the two EAto sites identified above. Mutation of both sites or of the E1 site alone in *cato2A *resulted in loss of GFP expression in Ch cells (Fig. 3G). It is notable that the E1 site is well conserved in *Drosophila pseudoobscura *(in fact it is an even closer match to the EAto consensus) whereas the E2 site differs markedly in the nucleotides flanking the core E box (Fig. 3A). Thus, it is highly likely that Ato directly regulates *cato *via the E1 binding site in the 2A enhancer. In contrast, *cato *is activated only indirectly by Sc in ES lineages. The identity of the factor(s) directly activating *cato *in ES lineages remains unknown, but it would presumably bind to the 2B enhancer (Fig. 3I).

The early Ch enhancer is also expressed in the *amos*-dependent dbd and dmd1 cells (Fig. 3B). These cells express both Amos and Ato, with the expression of Ato dependent on that of Amos [16]. Thus, *cato *could be a direct target of Amos, or an indirect target via activation of Ato. We find, however, that *cato *expression is not affected in these cells by the loss of *ato *function (Fig. 1E). In contrast, this expression is absent in *amos *mutant embryos (data not shown). Moreover, expression in *amos-*dependent cells is also absent when the E1 site is mutated (Fig. 3G). We conclude that *cato *is likely to be directly activated by both Ato (in Ch cells) and Amos (in dbd and dmd1 cells) via the same E box binding site (E1). This is the first identified direct target gene of Amos.

### *cato *mutation does not cause lethality

**Figure 4:**
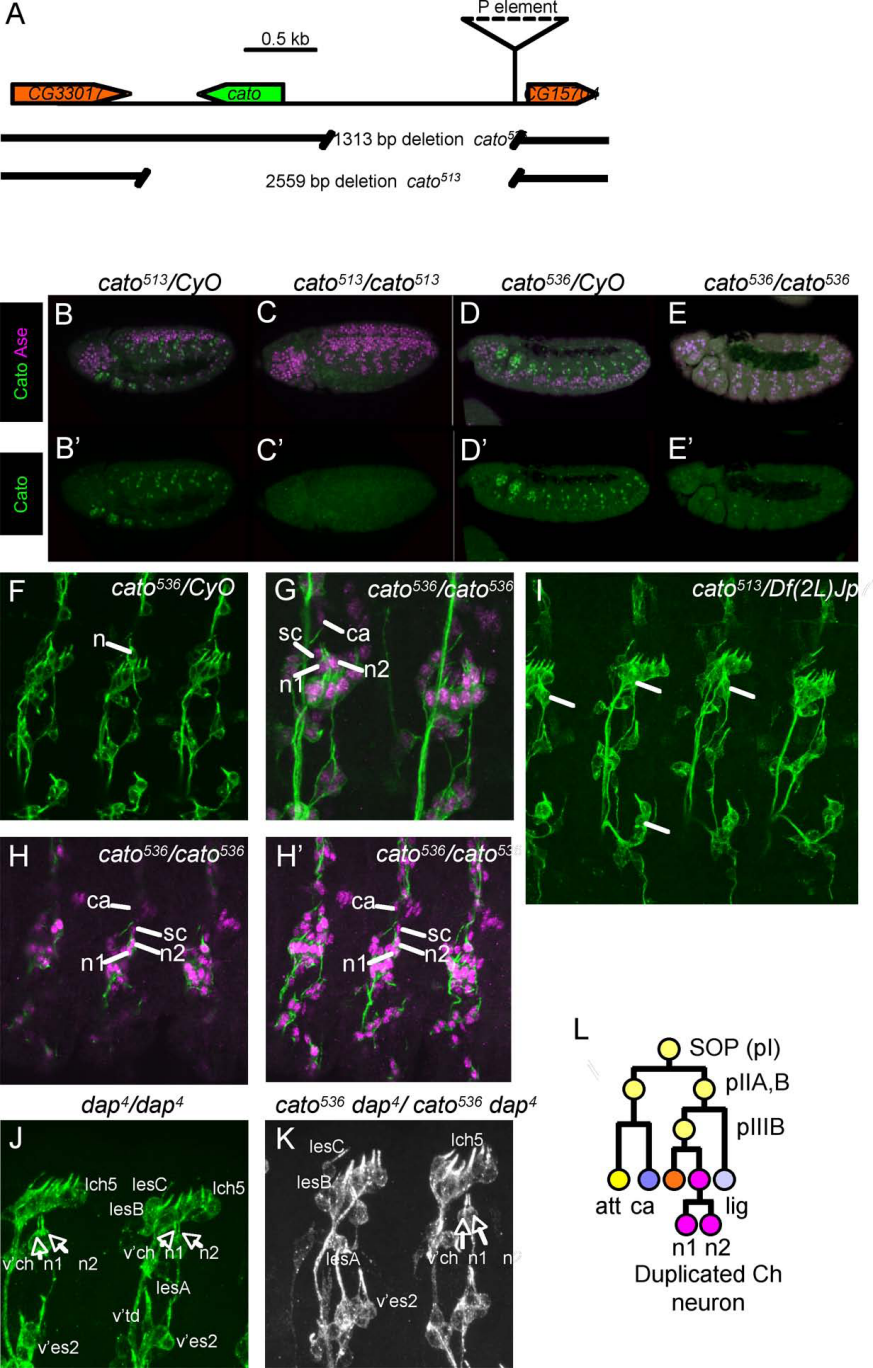
**Mutation of *cato *results in duplicated v'ch1 neurons**. (A) Schematic of *cato *genomic region showing the location of the P-element and excision deletions. (B-E) Expression of Cato protein (green) is absent or strongly reduced in *cato *mutant embryos (Ase expression shown in magenta) (B, B') Heterozygous *cato*^513^*/CyO *embryo. (C, C') Homozygous *cato*^513^*/cato*^513 ^embryo. (D, D') Heterozygous *cato*^536^*/CyO *embryo. (E, E') Homozygous *cato*^536^*/cato*^536 ^embryo. (F-K) Late stage embryos stained with 22C10 (green) for sensory neurons and anti-Cpo (magenta) for nuclei of neurons and support cells. Cells of the v'ch1 chordotonal organ are indicated: n = duplicated v'ch1 neurons; sc = unduplicated scolopale cells; ca = unduplicated cap cells. (F) Heterozygous *cato*^536^*/CyO *embryo, showing wild-type 22C10 pattern. (G, H) Homozygous *cato*^536^*/cato*^536 ^embryos, showing duplicated v'ch1 neurons (n1, n2). In (H) several confocal sections are shown. (I) Hemizygous *cato*^513^*/Df(2L)Jp7 *embryo, showing duplicated v'ch1 neurons. (J) Homozygous *dap*^4^*/dap*^4 ^embryo stained with 22C10, showing duplicated v'ch1 neurons. Selected other neurons are labelled, and show no obvious duplication. (K) Double homozygous *dap*^4^*cato*^536^*/dap*^4^*cato*^536 ^embryos stained with 22C10, showing similar phenotype to each single mutant in which v'ch neurons are duplicated but not adjacent neurons. (L) Schematic summary of the proposed neuronal duplication phenotype.

The fly stock, *KG07568*, contains a P-element insertion about 1.5 kb upstream of *cato *(Fig. 4A). To generate *cato *mutations, we mobilised the P-element to create imprecise excisions extending in the direction of the *cato *gene. Two deletions were generated named *cato*^536 ^and *cato*^513 ^. *cato*^513 ^is a protein null: the deletion removes the *cato *ORF and no Cato expression is apparent in homozygous *cato*^513 ^embryos (Fig. 4B, C). *cato*^536 ^deletes the *cato *upstream region but does not affect the transcription unit. Homozygous embryos show very little protein expression, particularly in the trunk (Fig. 4D, E). This is consistent with the loss of the major regulatory elements upstream of *cato*. Both mutations are viable, with little sign of gross defects. Adult flies appear normal and active. Thus, *cato *does not appear to play an essential role in gross neurogenesis.

### *cato *mutant embryos exhibit duplicated chordotonal neurons

Embryos homozygous for either *cato *mutation show a near normal arrangement of sensory neurons and support cells, as judged by MAb22C10 and anti-Cpo staining (Fig. 4 and data not shown). There is no clear evidence of differentiation or axon defects, in contrast to the phenotypes proposed previously based on the examination of chromosomal deficiencies [7]. A defect was observed, however, in the number of Ch neurons: embryos homozygous for either *cato *mutation exhibit frequent duplications of Ch neurons (Fig. 4F-I). For *cato*^536 ^mutant embryos, the v'ch1 neuron is duplicated in 84.5% of abdominal segments (n = 110). Of these, very few (1.8%) seem to be associated with duplicated scolopale cells with the remainder having a single scolopale cell (Fig. 4G, H). Cap cells are more difficult to identify with certainty, but in at least the majority of cases a single cap cell could clearly be associated with the duplicated neuron (Fig. 4G, H). Given this general lack of change in support cell numbers, we conclude that the duplicated neuron most likely results from a defect in cell division number rather than a disruption of asymmetric cell division or an increase in SOP formation (Fig. 4L). Ch neuron duplication is also seen for the vchA and vchB neurons, but only rarely for the lch5 neurons. Similar levels of neuronal duplication were observed in *cato*^513 ^mutant embryos, and the phenotype is present in embryos expressing a *cato *RNAi construct (PzL, data not shown), thus ruling out the possibility that it results from an effect of the deletion on the adjacent gene, *CG15704*.

We explored this neuronal duplication defect by looking for interaction with other genes involved in regulating the number of cell divisions that PNS neural precursors undergo. Cyclin A is known to be required for SOP cell divisions and it has been proposed that proneural factors regulate its expression by binding to an element in its first intron [24]. Loss of one copy of the *cyclin A *gene resulted in suppression of Ch neuronal duplication in *cato *homozygote embryos (55% of segments with duplicated v'ch1 neurons, n = 42). This finding supports the conclusion that *cato *mutants exhibit a specific proliferation defect in Ch neuronal lineages. *dap *encodes a CDK inhibitor that is required in the epidermis and CNS to terminate cell divisions at the appropriate time [12-14]. In *dap *mutants, cells typically undergo one extra round of division. We found that this is true for the PNS too: extra sensory neurons are present in *dap*^4 ^homozygous mutant embryos (Fig. 4J). Interestingly, not all sensory neurons are equally affected: the *dap *mutant phenotype closely resembles that of *cato *in that duplication appears most apparent for Ch neurons. In addition, *dap/cato *double homozygotes do not appear greatly different from either single mutant (Fig. 4K), suggesting that both genes function in the same pathway.

*cato *expression was shown above to persist particularly in Type II md neurons. These do not appear duplicated. Most of these are dendritic arborisation (da) neurons that have elaborately branched dendrites extending beneath the larval cuticle. We investigated whether differentiated da neurons showed morphological defects in *cato *mutants by examining dendrite branching patterns in third instar larvae. Da neuronal dendrites were detected using 22C10 (staining all da neuron dendrites) or Gal4^447^/UAS-mCD8-GFP (a class IV da neuron marker [25]). However, analysis showed no clear defects in the pattern or extent of branching of da neuron dendrites (data not shown).

### Redundancy between *cato *and *ato *in C1 precursor development

**Figure 5:**
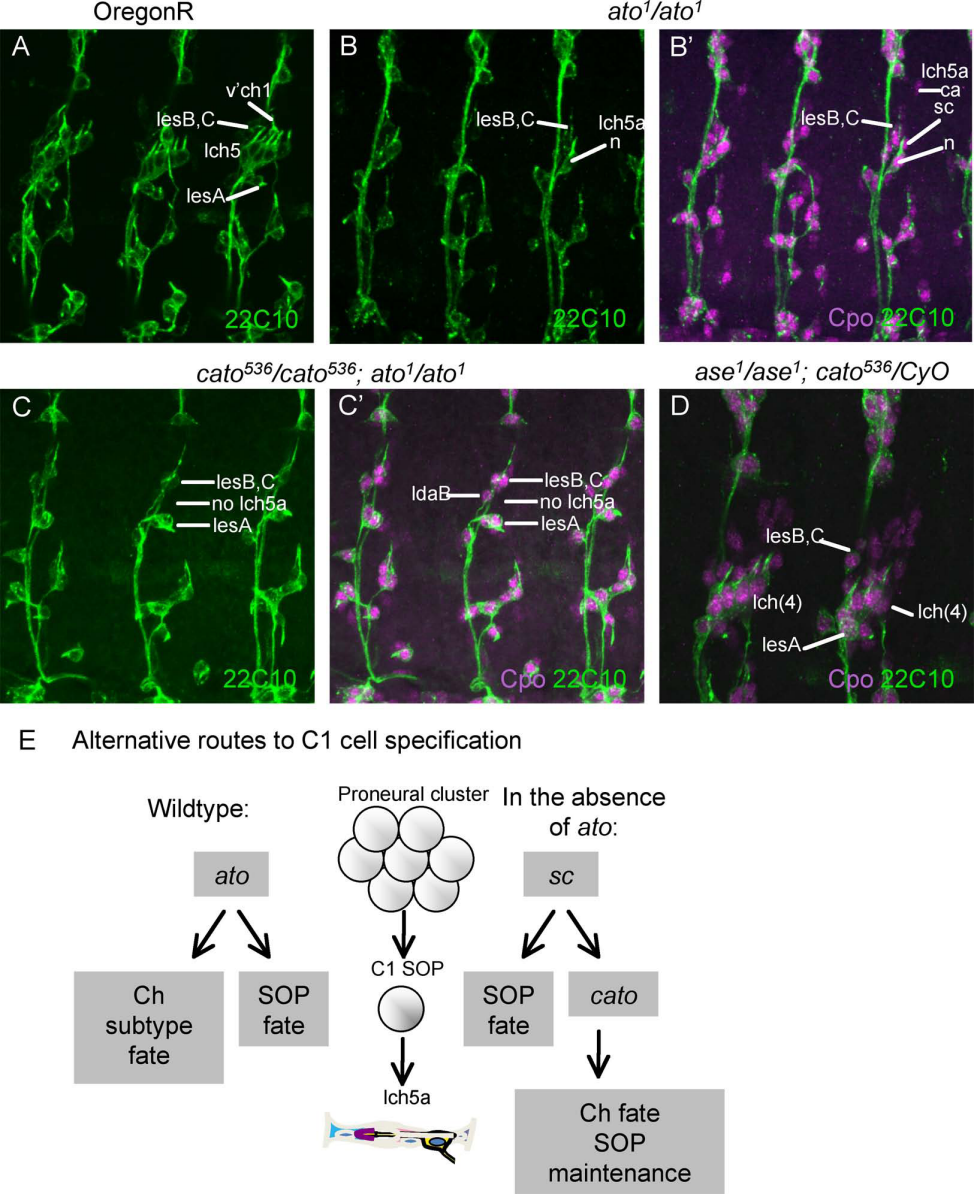
**Embryonic phenotype of double mutants**. (A-D) Abdominal segments from late stage embryos stained with 22C10 (green) and anti-Cpo (magenta in B', C', D). (A) OregonR (wild type) embryo with lateral Ch and ES neurons indicated. (B, B') Homozygous *ato*^1 ^mutant embryo, with neuron (B, B'), scolopale and cap cells (B, B') of lch5a indicated, as well as adjacent ES neurons. (C.C') Double homozygous *cato*^536^*; ato*^1 ^mutant embryo, with ES neurons visible but no lch5a organ. (D) Embryo homozygous for *ase*^1 ^and heterozygous for *cato*^536^, with lch5 Ch clusters reduced from five to four Ch organs. (E) Model for the dependence of C1 cell development on *cato*, *ato *and *sc*. See text for details.

In *ato *mutant embryos almost all Ch neurons are missing, but one neuron of the abdominal lch5 cluster usually still develops [19]. This is the anterior-most neuron of the cluster (lch5a) and it arises from C1, the first Ch SOP to appear. *cato *expression in C1 persists in *ato *mutant embryos (Fig. 1E). Therefore, although *cato *mutants do not exhibit loss of lch5 neurons (Fig. 4B, C), we speculated that *cato *might be responsible for C1 formation in the absence of *ato *function. To test this, we determined whether the lch5a neurons that remain in *ato *mutant embryos require *cato *function. In *ato*^1 ^homozygotes, 90.6% of abdominal segments contained an lch5 neuron (n = 53 segments) (Fig. 5A, B). In *cato*^536^; *ato*^1 ^double homozygotes, however, the presence of this neuron is strongly reduced (Fig. 5C): only 5% of segments contained a possible lch5 neuron (n = 40 segments). Thus, C1 (and the resulting lch5a neuron) can develop when either *ato *or *cato *are functional, suggesting at least some redundancy in their function for this cell.

### *ase *and *cato *cooperate in lch5 development

Like *cato*, *ase *is expressed after SOP selection. *ase *has roles downstream of SOP selection, but its phenotype in sensory lineages is mild considering its wide expression pattern[10]. One phenotype observed in *ase*^1 ^mutant embryos at a low frequency is the loss of one chordotonal organ from the lateral cluster of five organs (lch5>lch(4); 7% segments showing defect; n = 100 segments). We observed that the frequency of this lch(4) phenotype is greatly enhanced in *ase*^1 ^mutant embryos with one copy of *cato*^536 ^(28% segments showing defect (n = 100)) (Fig. 5D). Since support cells are missing in addition to the neuron, it appears that this phenotype results from an early defect in some Ch SOPs and is not linked to the later neuronal duplication defect of *cato *described above.

## Discussion

Although *cato *is a PNS-specific gene, its expression and function appear to be different in distinct lineages of the PNS. Its expression begins in Ch precursors just after their formation, but appears much later in ES lineages. Correlating with this pattern, we found that *cato *is directly regulated by *ato *in Ch SOPs but it is not a direct target of Sc in ES SOPs. This expression pattern, and its underlying regulation, appears to be characteristic of a number of genes, including the transcription factor Rfx and a number of its targets (S. Cachero, PzL, APJ, submitted). We refer to the pattern as 'Ch-enriched' and suggest that such genes mediate part of Ato's subtype specificity in neurogenesis. Interestingly, in different sensory lineages, it seems that *cato *is regulated by Amos and Ato through the same E box binding site.

The functions we have characterised for *cato *relate to its major site of expression: the Ch organs. The most obvious defect in *cato *mutant embryos involves supernumerary cell divisions in the neuronal branch of Ch lineages. This is reminiscent of the known roles of the other non-proneural bHLH proteins, *dpn *and *ase*. Thus, in the larval optic lobe, *dpn *expression maintains proliferation, whilst *ase *promotes cell cycle exit and neuronal differentiation [14]. The function of *cato *and *ase *in limiting cell division resembles the well-known function of vertebrate proneural-like bHLH factors in promoting the cell cycle exit of neuronal progenitors as a prelude to differentiation. This is opposed by HES factors (homologous to *dpn*), which maintain proliferation [1].

In the case of the larval optic lobe, *ase *functions in part via the CDK inhibitor, *dap *[14]. *dap *itself is generally required for cells to terminate cell division appropriately and cells generally undergo one extra division in *dap *mutants [12,13]. *dap *expression is highly dynamic in embryos [26], and it appears that a pulse of *dap *expression helps to ensure the timely shut down of cyclin function for appropriate cell cycle exit. We show here that *dap *is similarly required for Ch neurons. Moreover, the PNS phenotype of *dap *mutant embryos is strikingly similar to that of *cato*. This suggests that *cato *regulates *dap *in Ch neurons. Genetic analysis suggests this might be so, but we see no clear change in *dap *expression in *cato *mutant embryos (unpublished data). However, the complex and highly dynamic expression of *dap *may make small lineage-specific changes in expression difficult to detect. The idea that *cato *might regulate *dap *is consistent with previous observations that *dap *is under the control of multiple developmental regulators rather than of cell cycle regulators themselves [26,27], and also that *dap *is regulated by Ato in the developing eye [28]. *dap *is one of several cell cycle regulators (*cyclin E *[29] and *string *[30]) that have complex modular *cis*-regulatory regions. It is notable that *cato *appears to regulate only the division of the neuron and not support cells. We speculate that this division may require independent regulation from those of the support cells, because the number of neurons within a Ch organ varies in different locations, presumably as a result of extra neuronal cell divisions. For instance, some Ch organs in the adult femur have two neurons, whilst Ch organs in the antenna have three neurons [31].

The other functions detected for *cato *appear to be unrelated to the neuronal duplication function and show at least some redundancy with other bHLH regulators (*ato *and *ase*). In both these cases we suggest that *cato *plays a partially redundant role in maintaining SOP fate. In the absence of *ase *and *cato*, some Ch SOPs fail to form scolopidia. A similar situation applies to C1 in the absence of *ato *and *cato*. The apparent redundancy between *ato *and *cato *suggests that C1 SOP can form via alternative routes involving *ato *and *cato *(Fig. 5E). However, *cato *is expressed too late to be a proneural gene, and so another factor must supply the proneural function in the absence of *ato*. It seems likely that this factor is *sc*, which is expressed in C1 despite being the ES proneural gene [32]. Embryos with a mutation of the *achaete-scute *complex often show one missing scolopidium in the lch5 cluster [33], while *AS-C/ato *mutant embryos have no Ch cells at all. Such interchangeability of proneural functions between *ato *and *sc *is surprising since *sc *does not generally have the capacity to direct Ch subtype specification, as shown in misexpression experiments [3,34]. In contrast, *ato's *subtype specificity function is reflected in its ability to convert ES SOPs to a Ch fate [35]. We suggest that expression of *cato *in a *sc*-dependent C1 cell may provide sufficient subtype determination information when *ato *is absent. It is not clear why such a complicated exception should have arisen. One possibility is that C1 forms a unique neuronal type among Ch organs. Certainly there are a number of genes that are only expressed in, or are only absent from, this one neuron. For instance, MAb49C4 detects an antigen that is expressed in all lch5 neurons except lch5a [36]. Moreover, C1 appears to be functionally unique in that it acts to induce surrounding cells to differentiate as oenocytes via EGFR signalling [37,38]. This function of C1 appears to be 'rescued' by *cato *function, since the C1 cells present in *ato *mutants are able to recruit oenocytes [37].

Expression of Cato in ES lineages appears to be mainly as a prelude to late expression in the md/da neurons that derive from both ES and Ch lineages. As yet, no function has been discerned for this late expression, but we speculate that *cato *mutant larvae may exhibit a physiological defect in da neurons, which are thought to be required for nociception and thermoreception [39].

## Conclusions

Characterisation of the first mutations for *cato *has revealed roles in maintenance and cell division in Ch lineages. These roles are relatively subtle considering that *cato *is expressed widely in the developing PNS. Moreover, *cato *orthologues can be readily recognised among *Drosophila *species and other Diptera (unpublished observations), suggesting strong conservation. It is possible that further functions remain to be uncovered, perhaps in da neuron physiology or in the complex cephalic sense organs.

## Methods

### Fly stocks

Fly stocks used were *y*^*1 *^*w*^*67c23*^*; P{SUPor-P}KG07568 *[40], *ato*^*1 *^[41], w*; *wg*^*Sp1*^*/CyO*; *ry506 Dr*^*1 *^*P{Δ2-3}99B/TM6, Ubx *(Bloomington Stock Centre). They *cyclinA *stock was: w*; CycA^*C8LR1*^*/TM3, Sb*^*1*^*P{35UZ}2 *and the *dacapo *stock was *dap*^4^*/CyO, P{ftz/lacB}E3 *(both from Bloomington Stock Centre).

### Generation of anti-Cato antibodies

The Cato reading frame was subdivided and the two fragments were each cloned into the pGEX-2T expression vector. The reading frame for the first 100 amino acids, omitting the start codon, was amplified by PCR using the primers 5'-ATC**GGATCC**TACTACTCGTCTGCC-3' and 5'-GCGC**GAATTC**CGCTCAATCCAAATCC-3' (added restriction sites are/underlined). The primers for the remaining 89 amino acids were 5'-GGAC**GGATCC**CAGAAAAGGAGACGAC-3' and 5'-GTCA**GAATTC**CTGGACCGTGGGACTG-3'. Expression of GST fusion proteins was induced in *BL21(pLysS) *cells using 0.25 mM IPTG. After glutathione affinity purification, proteins were used to raise anti-Cato antibodies in sheep.

### Immunohistochemistry

Embryo antibody staining was carried out according to standard procedures. Primary antibodies used were sheep anti-Cato antibody (1:1000), mouse anti-22C10 and mouse anti-Elav (both 1:200; Developmental Biology Hybridoma Bank, Iowa City, Iowa), mouse and rabbit anti-GFP (1:500; Molecular probes, Invitrogen), rabbit anti-Cpo (1:500; [42]), guinea pig anti-Sens (1: 6250; [17] and rabbit anti-Ato (1:4000; [3]. The secondary antibodies (1:500) were from Molecular Probes (Invitrogen). Confocal microscopy was carried out using a Zeiss PASCAL and a Leica TCS SP2 microscope.

### P element imprecise excision

The P element insertion *P{SUPor-P}KG07568 *[40] was mobilised to create imprecise excisions that remove DNA between the insertion site and the *cato *gene. The *KG07568 *stock was crossed to a stock carrying transposase (D2-3). Male progeny containing the two elements were then mated individually to females from a *CyO *balancer stock. After mating, DNA was extracted from each male. DNA from pools of ten males was screened by PCR for deletions. If a shortened PCR product was detected, individual flies from that pool were screened further in individual PCR reactions. The PCR product was subsequently sequenced in order to establish the extent of the deletion. Deletion *cato*^513 ^was detected using primers P1 (GCTATCTATCGATGTGTAAGC) and 18R (TGTTATGTCCTC) and the smaller deletion, *cato*^536^, was detected using primer P1 and eCato2 (TTCACCGCCGTTCTGACC). These indicated deletions of 2559 bp and 1313 bp respectively.

### Reporter plasmid constructs

PCR amplified fragments were cloned into the pHStinger vector [43]. Genomic DNA fragments were amplified by PCR, and GFP reporter gene constructs were made in the transformation vector, pHStinger. These were used to make transgenic flies by microinjection into syncytial blastoderm embryos. In general, at least two transgenic lines were examined for each construct. Primers used were: *cato1.6k*: 5'-GCTGTATCAGGACACGAAGCTCC-3' and 5'-TTCACCGCCGTTCTGACC-3'. *cato1*: 5'-GTGGAGAAGTATTTGTCAG-3' and 5'-CTGCACCGACCCGACTTTG-3'. *cato2*: 5'-TCCAGGACCAAAGGC-3' and 5'-TCATTGCAGATCCGAGCG-3'. *cato2A*: 5'-GACTTTCACGCTCAACG-3' and 5'-TCATTGCAGATCCGAGCG-3'. *cato2B*: 5'-TCCAGGACCAAAGGC-3' and 5'-GTTGAGCGTGAAAGTC-3'. For site directed mutagenesis of E box sequence motifs, mutagenesis was carried out using the Quik-Change Site-Directed Mutagenesis Kit (Stratagene). Mutations induced were: E1: aacatatgg changed to aaaatattg; E2: agcatatgg changed to agaatattg.

## Authors' contributions

PzL co-designed and carried out the experiments and helped analyse the data. APJ co designed the experiments, analysed the data and wrote the paper. Both authors read and approved the final manuscript.
